# mAb-Functionalized Biomimetic MamC-Mediated-Magnetoliposomes as Drug Delivery Systems for Cancer Therapy

**DOI:** 10.3390/ijms241813958

**Published:** 2023-09-11

**Authors:** Francesca Oltolina, Maria del Carmen Santaella Escolano, Ylenia Jabalera, Maria Prat, Concepcion Jimenez Lopez

**Affiliations:** 1 Department of Microbiology, Faculty of Sciences, University of Granada, 18071 Granada, Spain; mcarmensantaella@correo.ugr.es (M.d.C.S.E.); yjabalera@ugr.es (Y.J.); cjl@ugr.es (C.J.L.); 2 Dipartimento di Scienze della Salute, Università del Piemonte Orientale “A. Avogadro”, Via Solaroli 17, 28100 Novara, Italy; maria.prat@med.uniupo.it

**Keywords:** magnetoliposomes, biomimetic magnetic nanoparticles, drug delivery systems, monoclonal antibodies, doxorubicin, target therapy

## Abstract

In cancer therapy, new therapeutic nanoformulations able to mediate targeted chemotherapy are required. Recently, biomimetic magnetic nanoparticles (BMNPs) mediated by MamC, a magnetosome protein from Magnetococcus marinus MC-1, have proven, in vitro and in vivo, to be effective drug nanocarriers (following the application of an external gradient magnetic field) and to allow combination with hyperthermia. However, these nanoassemblies require further optimization to improve cytocompatibility, stability and active targeting ability. Herein, we describe the production of the magnetoliposomes (LP) embedding BMNPs functionalized (or not) with doxorubicin (DOXO), [LP(+/−DOXO-BMNPs)], and their surface modification with the DO-24 mAb, which targets the human Met/HGF receptor’s ectodomain (overexpressed in many cancers). Nanoformulations were extensively characterized using TEM, DLS, FTIR and when tested in vitro, the lipid coating increased the colloidal stability and their biocompatibility, favoring the cellular uptake in cells overexpressing the cognate receptor. Indeed, the magnetoliposomes mAb-LP(+/−DOXO-BMNPs) exerted a specific active targeting ability by the presence of the mAb that preserved its immunocompetence. Both LP(BMNPs) and mAb-LP(BMNPs) were not toxic to cells, while +/−mAb-LP(DOXO-BMNPs) nanoformulations were indeed cytotoxic. Therefore, this study represents a proof of concept for the development of promising drug carriers for cancer therapy based on local chemotherapy directed by mAbs.

## 1. Introduction

Magnetic nanoparticles (MNPs) composed of magnetite (Fe_3_O_4_) have gained a great interest in biomedicine as a versatile platform that can be used as a drug nanocarrier for a directed chemotherapy as well as to mediate therapy combination, i.e., chemotherapy and magnetic hyperthermia [1]. This targeting is important because, despite the huge improvement in cancer treatments, conventional chemotherapy lacks specificity and selectivity, causing severe side effects. For instance, the chemotherapy agent used in the present study, doxorubicin (DOXO), is known for acting on myocardial cells, inducing cardiotoxicity. Moreover, as all the chemotherapy drugs, it induces DNA damage, resulting in cell cycle arrest and apoptosis also in healthy tissue [2].

Superparamagnetic iron oxide nanoparticles (SPIONs) achieved visibility for their inner properties and have been extensively used as nanocarriers for chemotherapy, and as contrast agents in magnetic resonance imaging (MRI) in theragnostic applications [3]. Moreover, their abilities to respond to an external magnetic field reaching the target of interest and being used as hyperthermia agents confer them a great potential in cancer therapy [4]. In this context, new promising nanocarriers have been identified in MamC-mediated biomimetic magnetic nanoparticles (BMNPs), offering advantages over traditional inorganic MNPs and iron oxide nanoparticles in general. BMNPs are produced inorganically in the presence of a MamC protein (magnetosome membrane-associated protein from *Magnetococcus marinus* MC-1) and in addition to their known superparamagnetic, biocompatible qualities and the fact they can be synthetized with sustainable, low cost and scalable procedures [5,6], they have two important advantages over synthetic MNPs: (*i*) the larger dimension of BMNPs (~40 nm) compared to most MNPs (≤30 nm) maximizes their magnetic moment per particle which increases the magnetic responsiveness when a gradient magnetic field (GMF) is applied [7,8] and (*ii*) MamC present in the external layers of BMNPs confers functional groups at the BMNP surface, enhancing the plasticity and the effectiveness of functionalization with no further unnecessary coatings which MNPs need [5]. Indeed, thanks to their surface/volume and their peculiar surface properties, BMNPs manage to bind different charged moieties (such as DOXO, oxaliplatin (Oxa) or α-choline kinase inhibitors) at physiological pHs via electrostatic interactions that can be weakened if the nanocompounds are at acidic pHs (typical in tumor microenvironments), facilitating the release of the absorbed molecule [5,6,9,10]. BMNPs coupled with these molecules have been tested in vitro and in vivo (DOXO-BMNPs) to mediate a local, directed chemotherapy combined with hyperthermia in localized tumors, demonstrating a better efficiency in tumor reduction compared to that achieved, under the same conditions, by systemic treatments [6,8]. However, some improvements of these nanoformulations are required to avoid an undesired release of the drug coupled to BMNPs, and to increase biocompatibility and colloidal stability.

Liposomes (LP) have been previously shown to be suitable candidates for the embedding of both MNPs [11] and BMNPs or BMNP-nanoassemblies [12,13]. Actually, the use of liposomes has been proposed since the 1970s, to ensure an efficient/stable encapsulation, solubilization of aggregating hydrophobic compounds, cargo protection from degradation, prolonged circulation and low liver accumulation [14]. Furthermore, in recent years and especially due to SARS-CoV-2, LP have been considered fundamental in delivering mRNA-based vaccines [15]. Various approaches have been evaluated to develop the best candidates for vaccine mRNA transfer, giving particular attention to the lipidic composition that could affect the delivery system. This procedure is critical not only in vaccine formulations, but also in order to embed NPs with a lipid layer. In fact, different protocol strategies have already been developed to generate a coating on magnetic nanoparticles using several lipids agents such as phosphatidylcholine (PC), palmitoyl-oleoylphosphatidylcholine (POPC), dimyristoylphosphatidylcholine (DMPC), distearoylphosphatidylcholine (DSPC), distearoylphosphoethanolamine (DSPE), dioleoylphosphocholine (DOPC), dipalmitoylphosphatidylcholine (DPPC), phosphatidylinositol (PI) and sphingomyelin (SM) [12,16,17]. The choice of the lipid and the protocol to produce the magnetoliposome is crucial to ensure, on the one hand, that the embedding does not shield the magnetic properties of the BMNPs needed for magnetic guidance/concentration for hyperthermia and, on the other, to maintain a size that allows the additional concentration of the nanoformulation in the tumor site by the enhanced permeability and retention (EPR) effects. In this context, Jabalera et al. [12] developed a protocol to embed BMNPs in unilamellar PC liposomes of ~150 nm. These authors demonstrated that the embedding did not affect the magnetic properties of BMNPs and improved their biocompatibility and colloidal stability. Garcia-Pinel et al. [13] also embedded Oxa-BMNPs in PC-unilamellar liposomes to reduce the Oxa-BMNPs’ interaction with the macrophages, which lead to toxicity and aggregation. These authors validate that the addition of both a lipid cover and further pegylation improved the biocompatibility and cellular uptake of the Oxa-BMNPs nanoassemblies.

Moreover, it is well known that the EPR effect is much more emphaticized when a targeting agent drives the active and specific interaction of the functionalized nanoformulations with the tumor cells [18,19]. In this scenario, several ligands such as growth factors (i.e., folic and hyaluronic acid) [20,21] and monoclonal antibodies (mAbs) directed against tumor-associated markers mainly overexpressed on cancerous tissue have been exploited in targeted cancer therapies [22,23]. In addition, transmembrane proteins can be considered biomarkers to be targeted and to be exploited in the field of immunotherapy, in which mAbs are not only used as targeting agents, but act as molecules able to activate the immune system [24]. For example, evidence of this strategy was developed by Chiang and collaborators that designed a brand-new drug delivery system that combines T-cell stimulators and checkpoint inhibitors with magnetic iron oxide NPs functionalized with the anti-CD3/CD28 and anti-PD-L1 antibody in a 4T1-metastatic lung carcinoma model [25].

Most of the biomarkers, considered tumor-associated markers, are surface receptors for growth factors and tyrosine kinase receptors (TKRs), which gained a lot of interest in targeting strategies. Particularly, Met, the tyrosine kinase receptor of hepatocyte growth factor receptor (HGF), is a well-known potential target since it is overexpressed in different types of cancer, as colon rectal carcinoma [26], glioblastoma [27] and breast carcinoma [28] and it has been successfully used as a targeting agent when coupled to MNPs [6,8,29]. There is evidence showing where mAbs were used in association with magnetoliposomes in different cancer models [30,31], paving the way for a feasibility study.

As a matter of fact, herein, we describe for the first time a DSPC liposome-based nanoformulation that embeds BMNPs functionalized with DOXO [namely, LP(DOXO-BMNPs)] and further functionalized at the surface with DO-24 mAb (directed against the ectodomain of the human Met) by means of a covalent bond. Thus, the aim of the current investigation is the designing of this nanoformulation that improves biocompatibility and colloidal stability compared to previous BMNPs-nanoassemblies and that also allows the active and specific targeting of the nanoformulations in cells overexpressing the receptor (GTL-16 cells). The nanoformulations were chemical–physically characterized to ensure the maintaining of their properties. In vitro biological tests were also performed to ensure the biocompatibility, immunocompetence and cytotoxicity of the DOXO-bearing nanoformulation. This proof of concept represents a step forward for the development of new promising approaches for cancer therapy involving a directed tumor targeting based on mAbs.

## 2. Results and Discussion

### 2.1. Synthesis and Characterization of the Nanoformulations

Iron oxide NPs are without any doubt a material with a great potential for applications in medicine. Besides their inner characteristics, a notable advantage is that their core sizes can be modulated, resulting in NPs with different sizes and dimensions that can be exploited for specific applications [32]. For instance, size and shape are fundamental parameters since it is reported that spherical-shaped Fe_3_O_4_ MNPs of about 73 nm caused eryptosis in red blood cells in vitro and induced oxidative stress in vivo [33].

**Figure 1:**
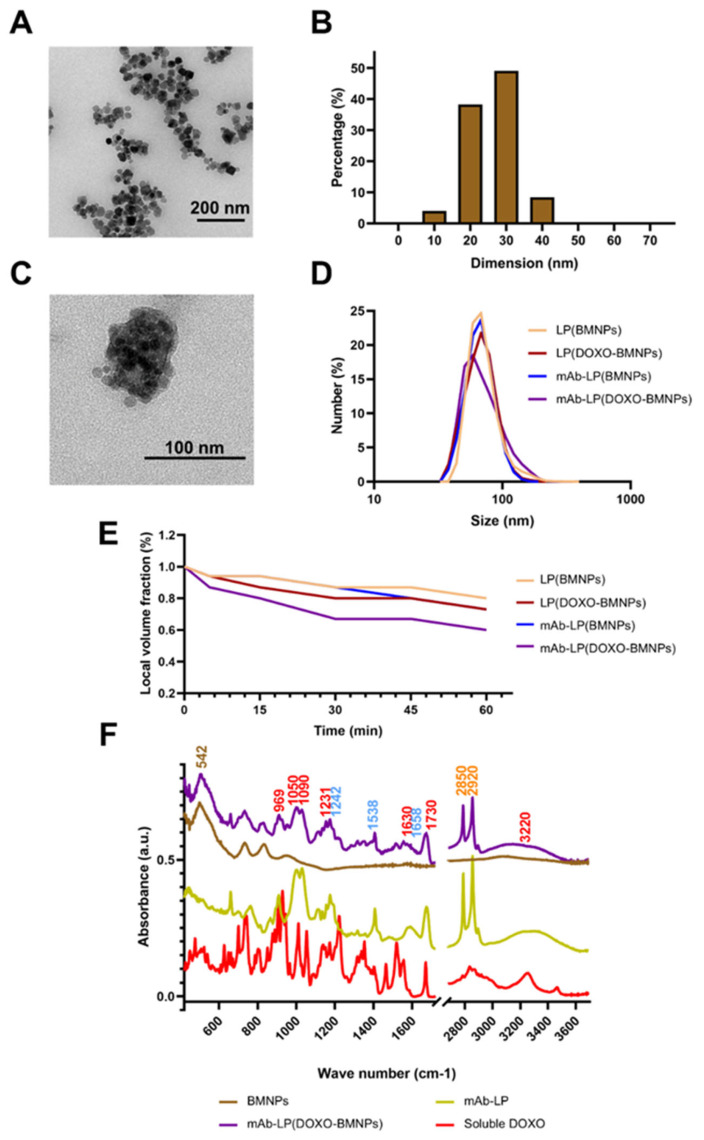
(**A**) Transmission electron microscopy images of biomimetic magnetic nanoparticles (BMNPs) and (**B**) size distribution. (**C**) Transmission electron microscopy image of a magnetoliposome. (**D**) Hydrodynamic size in number distribution for LP(BMNPs), LP(DOXO-BMNPs), mAb-LP(BMNPs) and mAb-LP(DOXO-BMNPs). (**E**) Colloidal stability of the nanoformulations at different time points. Height was normalized to its initial volume value as a function of time. (**F**) ATR-FTIR spectra of the BMNPs, mAb-LP, soluble DOXO and mAb-LP(DOXO-BMNPs). The signals corresponding to different elements are marked in red (DOXO), light blue (DO-24 mAb), orange (phospholipids) and brown (magnetite).

On the other hand, cubic MNPs do not exert massive cytotoxicity when tested in vitro and in vivo and, for this reason, a morphology of this type is to be preferred in medical applications [34]. TEM analysis of BMNPs showed their typical morphology as well-developed crystal faces with rhombic, rectangular, and square 2D morphologies. Particle size distribution averaged 35 ± 13 nm (Figure 1A,B).

The ability of the BMNPs of being functionalized without the use of chemical compounds is due to the presence of functional groups conferred by MamC protein. This is a fundamental advantage for these nanoplatforms since the addition of synthetic compounds, acting as linkers, could affect in a negative way the dimensions of the BMNPs, resulting in a reduced biocompatibility of the nanoformulations [34]. The amount of DOXO adsorbed on BMNPs was found to be 85 ± 0.26%, corresponding to 850 µg DOXO/5 mg of BMNPs. This load agrees with the one determined in previous couplings of BMNPs [6,8]. TEM micrographs of the LP(BMNPs) show magnetoliposomes of about 80 nm enclosing several BMNPs (Figure 1C). Dynamic light scattering (DLS) analysis proved that the hydrodynamic diameters of all nanoformulations were less than 100 nm (Figure 1D), confirming the data obtained at TEM. For instance, the size of the LP(BMNPs) and the LP(DOXO-BMNPs) were of 68.06 ± 3.4 nm and 58.77 ± 9.27 nm, respectively. Moreover, the presence of the mAb did not significantly increase either the size of nanoformulations since for mAb-LP(DOXO-BMNPs) was of 57.50 ± 5 nm.

Functionalization with the different moieties affected the colloidal stability, as shown in Figure 1E. LP(BMNPs) are the samples with the highest stability. This is consistent with the conclusions of other authors [12], which have observed that lipids improve the colloidal stability by reducing aggregation and also improve biocompatibility [35]. In addition, the polydispersity index (PDI) of the nanoformulations were 0.390 ± 0.016, 0.299 ± 0.011, 0.463 ± 0.038 and 0.379 ± 0.054 for LP(BMNPs), LP(DOXO-BMNPs), mAb-LP(BMNPs) and mAb-LP(DOXO-BMNPs), respectively, indicating a good dispersity of the samples.

The presence of DOXO and DO-24 mAb on mAb-LP(DOXO-BMNPs) nanoformulations at pH 7.4 was further confirmed by FT-IR analysis (Figure 1F). The FT-IR spectra shows absorption peaks characteristic of bonds present in the different elements that constitute the nanoformulation. In fact, the sample shows signals specific for DOXO: at 3220 cm^−1^ (typical of OH and NH), 1730 and 1630 cm^−1^ (for C=O), 1231 cm^−1^ (for C-O-C), 1090 cm^−1^ (C-O, tertiary alcohol), 1050 cm^−1^ (C-O, secondary alcohol) and 969 cm^−1^ (C-O, primary alcohol) [36]. It also shows signals for the mAb: at 1658, 1538 and 1242 cm^−1^, corresponding to the first, second and third amine, respectively [37]; and signals at 2920 cm^−1^ (asymmetric CH_2_) and at 2850 cm^−1^ (symmetric CH_2_) specific for the acyl chains of phospholipids [38]. Lastly, a representative peak at 542 cm^−1^ of the Fe–O bond in magnetite indicates the presence of BMNPs [23].

**Figure 2:**
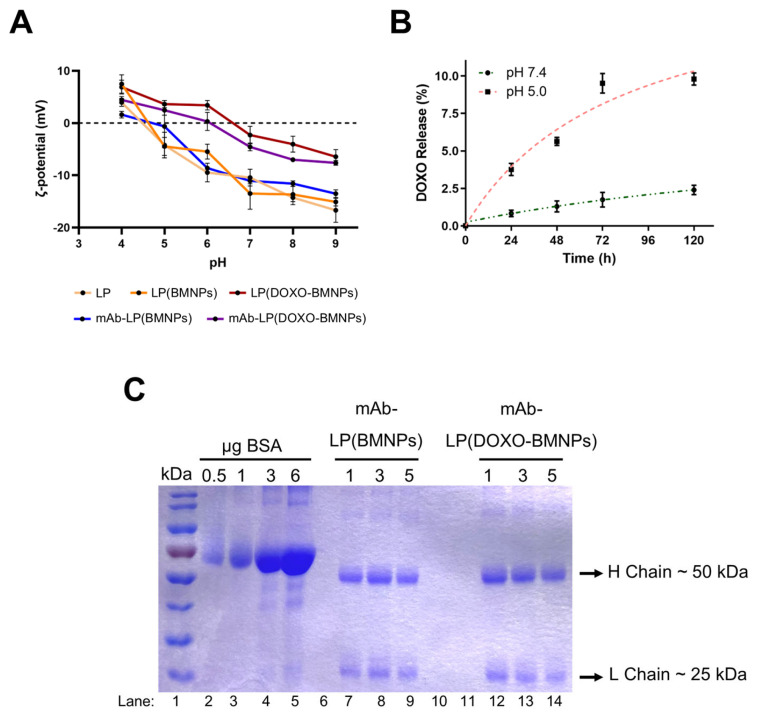
(**A**) ζ-potential of the different nanoformulations at different pHs. Stability of the nanoformulations: (**B**) DOXO release percentage from LP(DOXO-BNPs) during time and measured at physiological and acidic pHs (7.4 and 5.0). (**C**) DO-24 binding stability: figure of SDS-PAGE gel decorated with Coomassie blue. Lane 1 represents the marker used in the experiment; from lane 2 to 5 represented a standard curve of bovine serum albumin (BSA); from lane 7 to 9 are loaded mAb-LP(BMNPs) at 1, 3 and 5 days, respectively, as well as from lane 12 to 14 where the samples loaded are mAb-LP(DOXO-BMNPs) at the same time points.

At pH 7, the ζ-potential values indicate that all the nanoformulations were negatively charged. This is consistent with the enveloping in the DSPC liposomes, which exhibits a negative charge [39], indicating the success of the embedding (Figure 2A). This negative charge was also observed for other liposome compositions, probably due to the attaching of OH− on the –N(+)(CH_3_)_3_ group of the phosphatidylcholine present in the lipid film [13,40]. LP(BMNPs) displayed the highest negative charge at pH 7 (ζ~−13.48 mV), followed by mAb-LP(BMNPs) (ζ~−11.07 mV), mAb-LP(DOXO-BMNPs) (ζ~−4.56 mV) and LP(DOXO-BMNPs) (ζ~−2.30 mV). The fact that DOXO-bearing nanoformulations exhibited less negative charge may indicate a different interaction between the lipid and the BMNPs (or DOXO-BMNPs) and/or an incomplete covering of the DOXO-BMNPs nanoassemblies. However, this last option is not consistent with the TEM observations (Figure 1C).

The stability of DOXO-bearing nanoformulations and DOXO release at this pH value (7.4), mimicking the physiological conditions occurring in the blood stream, was tested (Figure 2B). At this pH value, the DOXO desorption pattern from magnetoliposomes was <2.5%, indicating the good stability of the nanoformulation. By comparing these data with those for DOXO release at this pH from the non-embedded nanoassembly DOXO-BMNPs (<4%; [6]), it can be concluded that the embedding increases the nanoformulation stability at the physiological pH value. Additionally, the stability of DO-24 in the mAb-bearing nanoformulations at physiological pH value was tested to ensure they could mediate active targeting. As shown in Figure 2C, the heavy and the light chains of the DO-24 mAb at 50 and 25 kDa, respectively, were detectable for up to 5 days for both mAb-LP(BMNPs) and mAb-LP(DOXO-BMNPs).

Therefore, these results anticipate that these nanoformulations may have the ability to be used as DDS, able to be guided both by the application of an external magnetic field [8] and by the mAb to a specific target, since the Fab fractions can recognize their target expressed by tumor cells. At an acidic pH, all the magnetoliposomes changed their surface charges becoming less negative than at a physiological pH (Figure 2A). mAb-bearing nanoformulations displayed at this pH a slightly positively charge because of the presence of the positively charged DO-24 (iep ~6.8, Prat, personal observations). The positive values for the ζ-potential of DOXO-bearing nanoformulations could be indicative of the DOXO release out of the magnetoliposome. In fact, it has been reported previously that, while BMNPs at pH 7 are negatively charged due to the negative functional groups of MamC, they become uncharged at pH 5, (iep of 4.4, [5]), thus favoring the release of the adsorbed DOXO [6,23]. These positive values could be indicative that DOXO has the ability to go through the magnetoliposome membrane once detached from the BMNPs, since magnetoliposomes are still intact at this pH value, according to TEM analyses (Figure 1C). To test this hypothesis, the stability of DOXO-bearing nanoformulations and DOXO release at pH 5 is measured, mimicking the acidic tumor microenvironment [41]. Our results show, in fact, a DOXO release from the magnetoliposome up to 10%. This release could provide a positive charge to the liposome, due to the exposure of the NH_3_^+^ groups of DOXO.

### 2.2. Cellular Interactions of Magnetoliposomes

**Figure 3:**
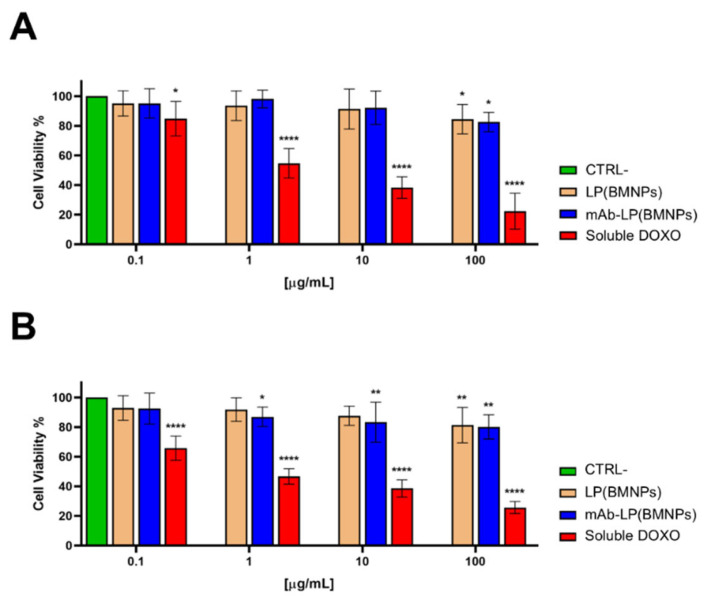
Cytocompatibility of the nanoformulations LP(BMNPs) and mAb-LP(BMNPs) on GTL-16 (**A**) and Huh7 (**B**) cells. Cell viability was performed by a MTT assay after incubation with different concentrations of the nanoformulations for 72 h. One-way ANOVA with Dunnett’s multiple comparison test was used to evaluate group differences (**** *p* 0.0001; ** *p* 0.001; and * *p* 0.05).

Cytocompatibility is one of the most important criteria for the nanoformulations to be validated before their eventual application in biomedicine [42]. BMNPs have been extensively reported to be biocompatible on several human cell lines originating from tumors of different origins [5,8,10,43], as are the magnetoliposomes embedding them [13]. Indeed, *naive* BMNPs do not induce ROS production in vitro up to 100 µg/mL or hemolysis in red blood cells [5,6]; in addition, the lipid coating present in magnetoliposomes increases the biocompatibility of the nanoformulations when incubated with white blood cells and RAW 264.7 macrophages up to 250 µg/mL for 12 h [13]. Herein, the cytocompatibility of the nanoformulations produced in the present study was tested on two carcinoma cell lines, the GTL-16 (human gastric carcinoma) and the Huh7 (human hepatocarcinoma) (Figure 3). GTL-16 (Figure 3A) and Huh7 (Figure 3B) cells were exposed to increasing concentrations of LP(BMNPs) and mAb-LP(BMNPs) (up to 100 µg/mL) for 3 days and their cytocompatibility was measured in a MTT assay. As expected, any of these nanoformulations exert significant toxicity on both cell types, even when treated at the highest concentration of the nanoformulations, since in all cases, >80% of the cells survived, thus demonstrating their high cytocompatibility.

The rationale behind a DDS is the use of an agent that specifically directs the drug or the compound to the site of interest. Various mAbs have been used to functionalize the BMNPs, thus mediating their specific interaction with different cell lines [6,23]; however, there are few studies in the literature where mAbs are conjugated to magnetoliposomes: Choi et al. [44] developed magnetoliposomes modified with antibodies against human epidermal receptor 2 (HER2) and they evaluated the mAb isolation efficiency on SK-Br3, a HER2-positive cancer cell, compared with HER2-negative HeLa cancer cells. In addition, Khaleghi et al. [19] synthetized magnetoliposomes functionalized with an anti-HER2 nanobody to increase image contrast and effectiveness in MRI applied to breast cancer cells. Dorjsuren et al. [45] combined photo-thermal therapy and targeted chemotherapy (cetuximab as the targeting agent and DOXO as the drug) in thermo-sensitive magnetoliposomes which represents a promising therapeutic strategy against breast cancer. The resulting nanoformulations had a diameter of about 120 nm and the coating with cetuximab increased the uptake of magnetoliposomes into the breast cancer cells. Moreover, the cellular viability was reduced when magnetoliposomes were carrying DOXO plus the addition of photo-thermal therapy using near-infrared (NIR) laser irradiation, indicating the effectiveness of the developed system in terms of targeting and drug efficacy.

**Figure 4:**
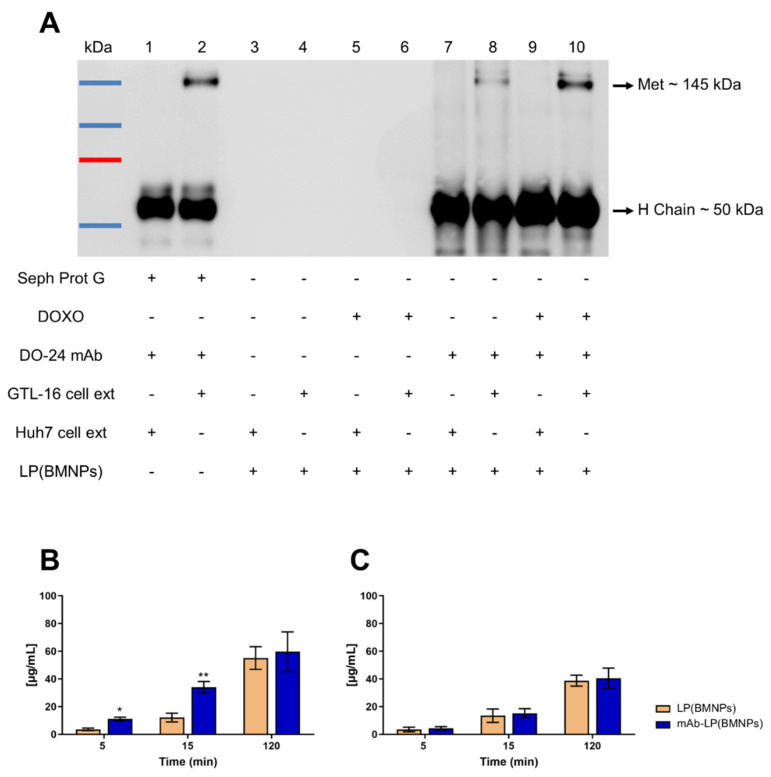
(**A**) Immunocompetence and specificity of LP(BMNPs) lanes 3 and 4, LP(DOXO-BMNPs) lanes 5 and 6, mAb-LP(BMNPs) lanes 7 and 8, mAb-LP(DOXO-BMNPs) lanes 9 and 10, analyzed in immunoprecipitation and SDS-PAGE/Western blot. The Met biomarker has a molecular weight of 145 kDa and it is immunoprecipitated only by mAb-functionalized nanoformulations from GTL-16 (Met^+^) cells, but not from Huh7 (Met^−^) cells. Moreover, the mAb heavy chain with apparent molecular weight of 50 kDa is detectable. (**B**,**C**) Histograms of the quantity of iron related to GTL-16 (**B**) and Huh7 (**C**) cells quantified with potassium thiocyanate. The results (expressed as mean ± SD) were obtained in three independent experiments made in triplicates. Two-way ANOVA with Sidak’s multiple comparison test was used to evaluate group differences (** *p* < 0.001, * *p* < 0.01).

So, immunoprecipitation experiments were executed to determine if DO-24 mAb linked to the nanoformulations maintained its ability in recognizing the cognate receptor. These experiments were performed by incubating the functionalized magnetoliposomes with detergent protein extracts obtained from cells overexpressing Met (GTL-16) or not (Huh7). Only magnetoliposomes carrying the moiety [mAb-LP(BMNPs) and mAb-LP(DOXO-BMNPs)] recognized the β chain of Met antigen solubilized from GTL-16 cells (Figure 4A, lanes 8 and 10), visualized as a 145 kDa band. This signal was not present in immunoprecipitates obtained by incubating these nanoformulations with extracts from Huh7 cells that do not express the Met receptor (Figure 4A, lanes 7 and 9), which indicates the preserved mAb activity when coupled on magnetoliposomes. Unfunctionalized nanoformulations (mAb-free) did not immunoprecipitate the antigen from the cells overexpressing it (Figure 4A lanes 4 and 6).

Whether or not the presence of DO-24 mAb improved the interaction of the nanoformulations with cells overexpressing (GTL-16) or not (Huh7) the pivotal receptor was tested (Figure 4B,C). When mAb-LP(BMNPs) were incubated with GTL-16 cells, iron internalization was higher at 5 and 15 min compared to that observed when these cells were incubated with LP(BMNPs), indicating that the interplay between the mAb and its target occurred at short periods of time (Figure 4B). These results are not observed in Huh7 (Met^−^) cells. In fact, no difference regarding iron internalization following upon cell treatment with LP(BMNPs) or mAb-LP(BMNPs) were observed (Figure 4C). At 2 h, the differences in iron internalization between the two treatments was not significant. This result is in accordance with previous observations that, at longer times of incubations, nanoformulations could exert some unspecific interactions, resulting in their internalization even in the absence of a receptor [6,46,47]. Nevertheless, the higher specificity for recognition showed at shorter times could be an advantage in a dynamic situation, such as that occurring in vivo. All together, these data confirm that the DO-24 mAb attached to the surface of magnetoliposomes specifically binds its receptor, showing that these nanoformulations can mediate active targeting.

**Figure 5:**
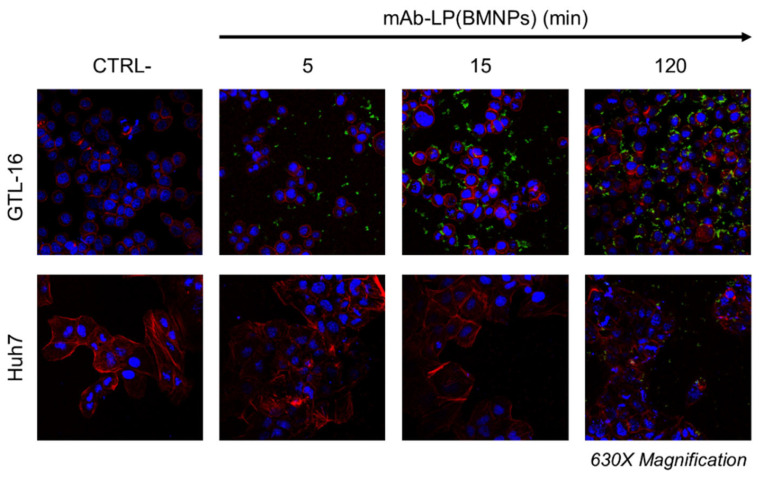
Interaction of mAb-LP(BMNPs) with cells. GTL-16 (Met^+^) and Huh7 (Met^−^) cells were incubated with mAb-LP(BMNPs) for 5 min, 15 min and 2 h at 37 °C and then stained: the green signal of the mAb loaded on LP(BMNPs) is revealed by secondary FITC-labeled antibodies and it is detected only in treated GTL-16 cells; the red signal matches the cytoskeletal actin (TRITC-phalloidin), while nuclei are stained in blue (TO-PRO-3). CTRL−, control untreated cells. Magnification 630×.

The interaction of mAb-LP(BMNPs) within cells was also assessed by tracking DO-24 mAbs in immunofluorescence experiments (Figure 5). The signal acquired by confocal microscopy for the FITC-conjugated secondary antibody was clear only when mAb-LP(BMNPs) were incubated with GTL-16 cells (top row), confirming the data obtained with iron quantification. The nanoformulations were mainly localized at the cell surface, but they were not internalized within cells. Lastly, a faint signal was only detectable when mAb-LP(BMNPs) were incubated on Huh7 cells for 2 h (bottom row). As expected, a low fluorescence intensity is also depicted when nanoformulations are incubated with Huh7 cells, confirming the data shown in the iron quantification experiments.

### 2.3. Enhanced Cytotoxicity of mAb-LP(DOXO-BMNPs)

It is well known that vesicles made by lipids not only improve the biocompatibility of the NPs that are embedded in, but also increase the therapeutic effect of active drugs providing their controlled release in order to extend the biological half-life or reducing toxicity of the compounds. Moreover, the possible advantage to use liposomes as delivering systems is due to their ability to entrap both hydrophilic and hydrophobic substances [48]. However, the thin-layer hydration method used to obtain liposomes could result in a low encapsulation efficiency of the drug and this problem can be solved coupling the chemotherapy agent directly to the BMNPs allowing its prolonged circulation and its activity.

**Figure 6:**
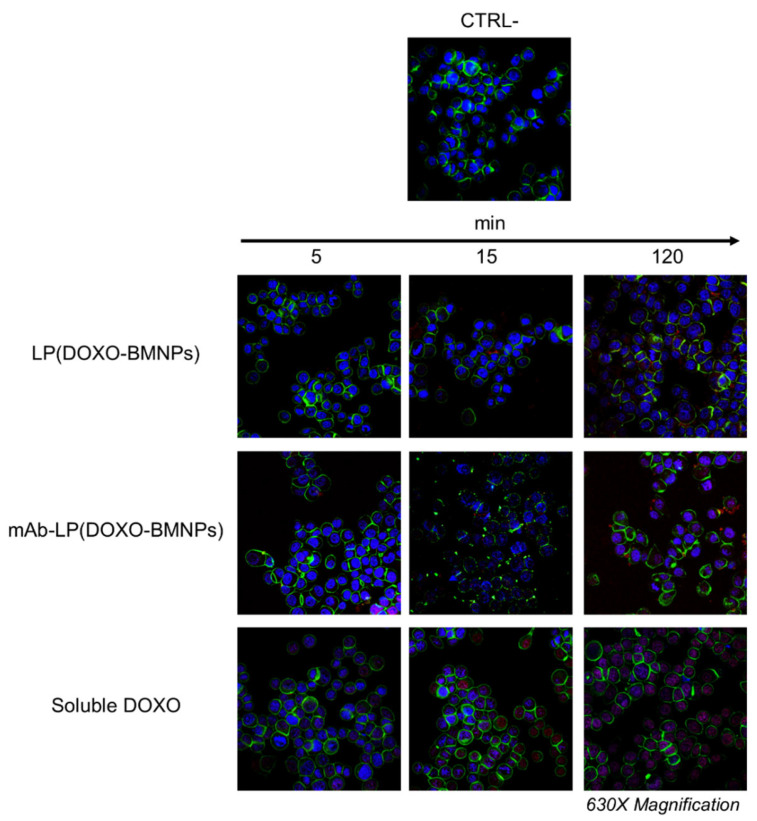
The presence of the mAb enhances the cellular uptake of Doxorubicin (DOXO) coupled to LP(BMNPs). GTL-16 cells were incubated at 37 °C with 100 µg/mL of +/−mAb-LP(DOXO-BMNPs) for different times (from 5 to 120 min). Soluble drug was used as a positive control. Cells were fixed, permeabilized, stained for cytoskeletal actin with fluorescein isothiocyanate (FITC)-phalloidin (green) and for nuclei with TO-PRO-3 (blue) and visualized at confocal microscopy. DOXO was detectable for its intrinsic fluorescence in red. CTRL−, control untreated cells. Magnification 630×.

In another set of experiment, the fate of the drug was analyzed when DOXO-bearing nanoformulations [LP(DOXO-BMNPs) and mAb-LP(DOXO-BMNPs)], as well as the soluble drug, were incubated with GTL-16 cells for different periods of time (Figure 6). In this case, cytoskeletal actin was detected as the green signal due to the FITC-phalloidin, while the red fluorescent signal was due to the inner properties of DOXO. When cells were incubated with soluble DOXO (Figure 6, bottom row), its signal was visible in the cell nuclei and its intensity increased in a time-dependent manner. When GTL-16 cells were exposed to LP(DOXO-BMNPs) and mAb-LP(DOXO-BMNPs), the DOXO signal was also observed in the cell nuclei in all cases, but some differences in the DOXO delivery were observed due to the presence of the DO-24 mAb. In the experiments performed with LP(DOXO-BMNPs), the intensity of the DOXO signal in the nuclei was inferior to that of the soluble DOXO. On the contrary, when cells were incubated with mAb-LP(DOXO-BMNPs), the DOXO signal in the cell nuclei was much more intense (Figure 6, middle row). This result agrees with those in Figure 5, indicating that the presence of the mAb results in a more specific interaction that ends in an enhanced DOXO cellular uptake.

**Figure 7:**
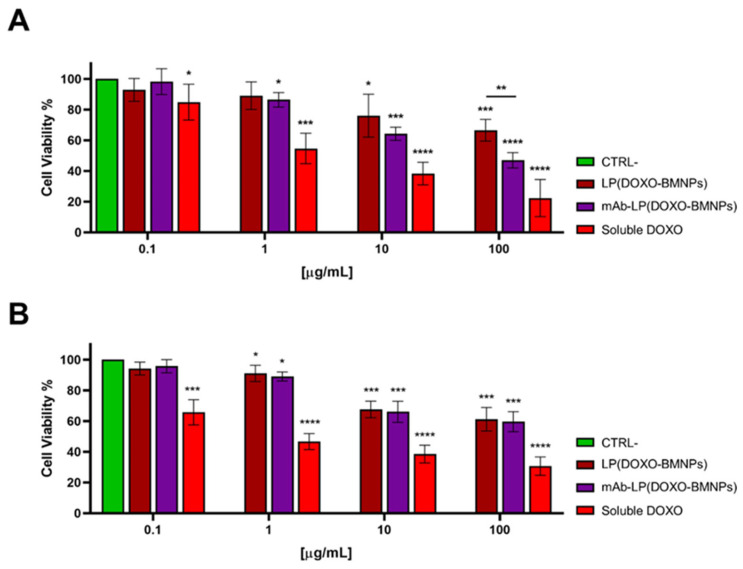
Cytotoxicity of the functionalized nanoassemblies on GTL-16 (Met^+^) (**A**) and Huh7 (Met^−^) (**B**) cells. Cell viability was performed by a MTT assay after incubation with different concentrations of the nanoformulations for 72 h. Two-way ANOVA with Dunnett’s multiple comparison test was used to evaluate group differences (**** *p* < 0.0001; *** *p* < 0.001; ** *p* < 0.01; and * *p* < 0.05).

Moreover, the toxic activity of +/−mAb-LP(DOXO-BMNPs) was assessed by a MTT assay after 3 days of incubation of the two nanoformulations with GTL-16 and Huh7 cells. As observed in Figure 7, the highest cytotoxicity was exerted by soluble DOXO in both cell lines and in a dose-dependent way, confirming data reported in literature [6,49]. In any case, the presence of the DO-24 mAb coupled to the nanoformulations increased their toxicity on GTL-16 cells (Figure 7A), while no significant effects were reported on Huh7 cells (Figure 7B). In detail, the most interesting difference was noted at the dose of 100 µg/mL where mAb-LP(DOXO-BMNPs) exerted a significant toxicity on GTL-16 cells (46.93% cell death) compared to that exerted by LP(DOXO-BMNPs) (66.51%). By comparing these data to the results described previously [6] using non-embedded mAb-DOXO-BMNPs, it seems that the embedding reduces the activity of the nanoformulations, probably associated to the slower DOXO release (Figure 2B). In any case, the data obtained confirm that the nanoformulations designed here could be candidates for a selective (ligand mediate) and directed DOXO delivery system whereby they are cytocompatible in the absence of the drug but become cytotoxic when DOXO is involved.

## 3. Materials and Methods

### 3.1. Production of BMNPs in Presence of Recombinant MamC protein

The expression and the purification of MamC protein was obtained following the protocol previously described by Valverde et al. [7]. Shortly, transformed *Escherichia coli* TOP10 cells (Life Technologies: Invitrogen, Grand Island, NY, USA) were allowed to grow at 37 °C in Luria–Bertani (LB) broth with the addition of ampicillin (0.1 mg/mL) and isopropyl-D-thiogalactopyranoside (IPTG, Fisher BioReagents, Pittsburgh, PA, USA) was used to induce the expression of the MamC protein. MamC purification was performed in denaturing conditions (6 M urea) by using a HiTrap chelating HP column (GE Healthcare, Chicago, IL, USA) in an ÄKTA Prime Plus FPLC System (GE Healthcare, Chicago, IL, USA). Lastly, the selected fractions which contain MamC were gradually refolded at 4 °C through dialysis. The obtained protein was then used in the biomineralization process. Following the process previously presented in [6,7,8], the synthesis of BMNPs took place in anaerobic conditions inside an anaerobic Coy chamber (96% N_2_/4% H_2_, Coy Laboratory Products, Grass Lake, MI, USA) at 1 atm total pressure, 25 °C and a pH value of 9. Once produced, the solid precipitates were incubated for one month and then, by a magnetic concentration, extensively washed with deoxygenated Milli-Q water. At the end, BMNPs were resuspended in oxygen-free water, sterilized, and used for further experiments.

### 3.2. Functionalization of BMNPs with Doxorubicin

BMNPs were coupled with the chemotherapeutic agent doxorubicin (DOXO) to obtain the nanoassembly DOXO-BMNPs, following the same procedure described previously [6,8]. In detail, BMNPs were mixed with DOXO in a ratio 5:1 and resuspended in 0.01 M HEPES [4-(2-hydroxyethyl)-1-piperazineethanesulfonic acid], 0.15 M NaCl (so-called binding solution) inside hermetically closed Eppendorf tubes to prevent the deterioration of magnetite. Nanoformulations were maintained in rotation on a rotary mixer (Richmond Scientific, Chorley, LANCS, UK) for 16 h at room temperature. At the end of the incubation, the solid components were collected with a neodymium magnet and washed three times with the binding solution and the pool of the supernatant (unbound DOXO) and the washings were used to determine the content of unbound DOXO, which was indirectly measured by UV-Vis spectroscopy (λ = 495 nm) (NanoDrop2000–Thermo Scientific, Waltham, MA, USA) until the measured optical density was <0.02 (background signal). The percentage of bounded DOXO (bD%) was calculated from the absorbance measurements (Abs), following the following equations:

Abs bDOXO=Abs SS DOXO−Abs ubDOXO

$$\mathrm{bD}\%=\frac{\mathrm{Abs}\;\mathrm{bDOXO}}{\mathrm{Abs}\;\mathrm{SS}\;\mathrm{DOXO}}\times 100$$

Abs bDOXO, Abs SS DOXO and Abs ubDOXO are the absorbance values of the bounded DOXO, DOXO stock solution (1 mg/mL) and the supernatants/washings collected after the coupling, respectively. After functionalization, the DOXO bound to the nanoformulations was calculated to be 85% ± 0.26, corresponding to 850 µg DOXO coupled to 5 mg of BMNPs.

### 3.3. Synthesis of Magnetoliposomes and Their Functionalization with a Monoclonal Antibody

The method described by Garcia-Pinel et al. [13] was adapted for the synthesis of the BMNPs encapsulated in liposomes, LP(BMNPs) as well as to produce liposomes encapsulating DOXO-functionalized BMNPs, further functionalized with the monoclonal antibody, mAb-LP(DOXO-BMNPs). Briefly, 5 mg of 1,2-distearoyl-sn-glycero-3-phosphocholine (DSPC) (Avanti Polar Lipids, Alabaster, AL, USA) were dispersed in 5 mL chloroform which was subsequently eliminated by using vacuum rotatory evaporation (Buchi, Rotavapor-R, Flawil, SG, Switzerland). The thin film layer was hydrated at 37 °C with an appropriate volume of ultrapure water containing 5 mg of BMNPs or DOXO-BMNPs and left shaking at 220 rpm for 1 h.

The DO-24 monoclonal antibody (mAb), belonging to the IgG 2a/k class and produced against the ectodomain of the human Met/HGF receptor tyrosine kinase [50,51], was used for the further functionalization of the magnetoliposomes to produce the nanoformulations mAb-LP(BMNPs) and mAb-LP(DOXO-BMNPs). Prior to the experiment, DO-24 mAb was activated with Traut’s reagent, as reported [52,53]. Briefly, 2 mg/mL Traut’s reagent was added to 1 mg/mL mAb and left for 1 h at 37 °C. Subsequently removing the extra reagent, the sample was centrifuged at 10,000 rpm and 21 °C for 5 min with a 30 kDa centricon (Millipore, Burlington, MA, USA) and stored.

The nanoformulations mAb-LP(BMNPs) and mAb-LP(DOXO-BMNPs) were produced by following a two-step protocol. Firstly, mAb-liposomes (mAb-LP) were produced by attaching mAb covalently to the liposomes. To this goal, liposomes were prepared by mixing 1 mg PEG 2000 and 76 µg DSPE-PEG 2000 Maleimide in 5 mL chloroform, which was then removed as described above. The thin film layer was hydrated at 37 °C with 1 mL of ultrapure water and left shaking at 37 °C, 220 rpm for 1 h. The sample was then transferred to an Eppendorf tube and 1 mg/mL of activated DO-24 was added and incubated on a rotary shaker for 24 h to obtain mAb-LP. The antibody not attached to the liposomes was removed by filtration with a 30 kDa centricon (Amicon) at 4000× *g* for 3 min at 21 °C. The quantity of the mAb was measured by UV-Vis spectroscopy (λ = 280 nm, NanoDrop2000, Thermo Scientific, Waltham, MA, USA). The final amount of DO-24 was calculated from the differences between the concentrations of the mAb in solutions before and after the functionalization with the liposomes. After the functionalization, the mAb covalently bound to LP was calculated to be 50% ± 0.19 corresponding to 500 µg per sample. Secondly, DSPC liposomes were prepared as described above; with the modification consisting of the thin lipid layer was then resuspended with 1 mL of BMNP or DOXO-BMNPs (5 mg/mL) with the mAb-LP complexes in ultrapure water at 37 °C. The entire resulting sample was shaken for 1 h at 220 rpm and 37 °C, then sonicated for up to 15 min at 37 °C and stored at 4 °C until use.

All the reagents used for the sample’s preparation described in the paper are reported in Table 1 below.

### 3.4. Chemical–Physical Characterization of the Magnetic Nanoformulations

BMNPs and LP(BMNPs) were analyzed by using a STEM Philips Model CM20 microscope. To this goal, samples were embedded in Embed 812 resin and sections of about 50–70 nm were prepared with Reichert Ultracut S microtome (Leica Microsystems GmbH, Wetzlar, Germany). BMNPs size was measured on ~1000 nanoparticles per experiment using ImageJ 1.48 v software (ImageJ, Maryland, MD, USA).

Hydrodynamic radius of the different nanoformulations was determined by dynamic light scattering (DLS) at physiological pH and the ζ-potential analyses were performed at pH values ranging from 4 to 9, at 25 °C in a Zetasizer Nano ZS instrument (Malvern Instruments, Instruments Ltd., Malvern, Worcs, UK). For these analyses, an amount of each sample was added to oxygen-free NaClO_4_ 10 mM, then the pH value was straight settled. To prevent aggregation, samples were firstly sonicated for 5 min and then promptly measured (nine replicas per sample).

FTIR spectrometer (model 6600, Jasco, Tokio, Japan) equipped with an attenuated total reflection (ATR) diamond crystal window (ATR ProOne) was used to perform Fourier-transform infrared (FTIR) analysis. The spectrum of each sample measured in the wavenumber ranged from 4000 to 400 cm^−1^, at 2 cm^−1^ resolutions.

The sedimentation process of the various nanoformulations was used to determine their colloidal stability, as formerly stated by Jabalera et al. [23]. An aliquot of 0.4 mL of 30 mg/mL of each nanoformulation was agitated thanks to a vortex for 1 min (corresponding to the time zero of the experiment) and allowed to settle. The phase separation was recorded at various intervals of time up to 1 h to determine complete sedimentation of all the nanoformulations.

The stability of LP(DOXO-BMNPs) nanoformulations at pH 7.4 and pH 5 was determined, as reported by Oltolina et al. [29]. LP(DOXO-BMNPs) were incubated from 1 to 5 days under constant stirring (200 rpm) at 37 °C, both in phosphate buffered saline (PBS)—pH 7.4 added with of 10% foetal calf serum (FCS) and in citric acid solution—at pH 5. Three replicas were performed for these experiments. At a certain time-interval, the nanoformulations were collected by a magnet and the supernatants were analyzed by UV-Vis spectroscopy. PBS + 10% FCS and citric acid buffers were used as controls for the relevant set of samples. The DOXO concentration in the supernatants at each time was measured, and the released amount of DOXO from the nanoformulations was indirectly calculated from these measurements and expressed as a percentage of the amount that was adsorbed on BMNPs at the beginning of the test.

The stability of the mAb-functionalized nanoformulations was evaluated directly on mAb-LP(BMNPs) and on mAb-LP(DOXO-BMNPs). The nanocompounds were magnetically recovered and washed, boiled in Laemmly Buffer and run in 10% sodium dodecyl sulfate-polyacrylamide gel electrophoresis (SDS-PAGE), as detailed in ref. [6]. Coomassie Blue staining was used to determine the presence of the mAb coupled to the nanoformulations.

### 3.5. In Vitro Biological Characterization and Cellular Interactions of the Magnetic Nanoformulations

#### 3.5.1. Cell Lines

Huh7 [54], a well-differentiated hepatocyte-derived cell carcinoma negative for Met/HGFR expression, and GTL-16 [55], a weakly differentiated human gastric carcinoma-derived cell line which expresses Met/HGFR, were cultured in Dulbecco’s modified Eagle’s medium (DMEM, Sigma-Aldrich, St. Louis, MO, USA), supplemented with 10% FCS, 50 µg/mL streptomycin and 50 U/mL penicillin (so-called complete medium). Cells were incubated at 37 °C, 5% CO_2_ and split at ratios 1:6 and 1:3 for Huh7 and GTL-16, respectively, when at 90% to 95% confluence.

#### 3.5.2. MTT Assay

Cells (≈12 × 10^3^ and 6 × 10^3^ for GTL-16 and Huh7, respectively) were seeded in 96-well plates for 1 day. After that, cells were treated with various amounts of nanoformulations (from 0.1, up to 100 µg/mL) in 100 µL of complete medium, as well as equimolar amounts of soluble DOXO, normalized for the quantity of the compound coupled to BMNPs. Following a 3-day incubation period at 37 °C and 5% CO_2_, the viability of the cells was assessed using the MTT colorimetric test, as previously mentioned [29]. Briefly, 20 µL of MTT solution (5 mg/mL dispersed in PBS) was added to each well and incubated at 37 °C for 2 h before the removal of the supernatants. The formazan crystals were eventually dissolved using a solution made of HCl (0.2 N) in C_3_H_8_O, and the optical density was read with a 570 nm wavelength using a Victor microplate reader (PerkinElmer, Waltham, MA, USA). The measure of the absorbance of untreated cells reflected the 100% vitality, while results obtained from cells that had undergone various treatments were compared to this value. Each experiment was repeated for no less than five times.

#### 3.5.3. Immunocompetence Validation of mAb-Functionalized Magnetoliposomes

The immunocompetence of the mAb-LP(BMNPs) was evaluated by incubating this nanoformulation and LP(BMNPs), used as negative control, with protein extracts prepared from GTL-16 cells and Huh7 in DIM buffer (50 mM PIPES pH 7.4, 300 mM saccharose, 100 mM NaCl, 5 mM EGTA, 5 mM MgCl_2_ and 100 µM ZnCl_2_), 1% Triton X-100, 1 mM TRIS HCl pH 8.8 and a cocktail of protease inhibitors at 4 °C overnight, as described [56]. +/−mAb-LP(BMNPs) were washed three times by magnetic decantation, then proteins were solubilized, warmed at 95 °C and separated in 10% SDS-PAGE. Polyvinyl fluoride (PDVF) membranes for Western blot were used for the transfer of the samples and stained with anti-Met DQ-13 mAb, as already reported [57]. After that, the membranes were reacted with horseradish peroxidase-conjugated affinity purified rabbit anti-mouse Ig antibodies (RaMIg/PO, 1:5000), reacted with enhanced chemiluminescence (ECL) and analyzed in the Versadoc device (Biorad, Hercules, CA, USA).

#### 3.5.4. Iron Quantification by Potassium Thiocyanate

Iron content within cells was measured as described previously [58]. Briefly, cells (≈50 × 10^4^ and 25 × 10^4^ for GTL-16 and Huh7, respectively) were seeded in 6-well plates and incubated as reported above. The day after, cells were treated with a fixed amount (100 µg/mL) of +/−mAb-LP(BMNPs) suspensions in complete medium. At distinct timings (5, 15, 120 min) the supernatant was removed and after being washed two times with fresh PBS and trypsinized, cells were transferred into 0.5 mL Eppendorf tubes and centrifuged at 1500 rpm for 5 min. A solution of 37% HCl, mixed with 10% H_2_O_2_, was used to dissolve cellular pellets after an incubation of 20 min at 25 °C. Then, samples were reacted with 1 mL of 1% potassium thiocyanate in Milli-Q water, and their absorbance values were measured at 490 nm. The absorbance from a standard curve created using the same procedure was used to calculate the concentration of ferric ions in the samples. The treated samples had their endogenous iron removed, and the untreated control cells served as normalizer. A minimum of three experiments were conducted.

#### 3.5.5. DOXO Internalization in Cells

Cells (≈24 × 10^3^ GTL-16 per well or 12 × 10^3^ Huh7 per well) were seeded on glass coverslips in 24-well plates and, the day after, a fixed amount (100µg/mL) of +/−mAb-LP(DOXO-BMNPs) suspensions in complete medium was added to cells. The nanoformulations were incubated for different time points (5, 15, 120 min) as well as an amount of soluble DOXO (as a positive control) normalized for the one loaded on +/−mAb-LP(DOXO-BMNPs). A solution of paraformaldehyde (4 wt%) was used to fix the cells seeded on the coverslips after being rinsed with fresh PBS pH 7.4. Cells were washed in Tris-Buffered Saline (TBS) with 5% Bovine Serum Albumin (BSA), 0.1% Triton X-100 and 5% goat serum to reduce unspecific interactions before being stained and permeabilized [57]. TRITC-phalloidin (1:300, Sigma-Aldrich, St. Louis, MO, USA—excitation at 543 nm; emission at 560–620 nm) or FITC-phalloidin (Sigma-Aldrich, St. Louis, MO, USA—excitation at 488 nm; emission at 500–535 nm) was used to stain cytoskeletal actin or mAbs were detected with FITC-labeled rabbit-anti-mouse IgG (1:500, Abcam, Cambridge, Cambs, GB—excitation at 488 nm; emission at 500–535 nm) and nuclei with TO-PRO-3 (1:1000, Life Technologies, Carlsbad, CA, USA—excitation at 633 nm; emission at 650–750 nm). DOXO was identified after excitation at 476 nm and emission at 575–630 nm. Fluorescence was detected with a Leica TCS SP2 AOBS Spectral Confocal Scanner microscope (630×). ImageJ 1.48 v software (ImageJ, Maryland, MD, USA) was used for analysis.

### 3.6. Statistical Analysis

Data are presented as mean ± standard error of at least three duplicate measurements. The statistical analyses were carried out using GraphPad Prism version 10 for Windows, GraphPad Software (GraphPad Prism, San Diego, CA, USA), with a one-way ANOVA and a Dunnet’s multiple comparisons test for grouped analyses. When the *p* values were *p* 0.05 (*), *p* 0.01 (**), *p* 0.001 (***) and *p* 0.0001 (****), the statistical differences between the treatments were deemed significant.

## 4. Conclusions

The outcomes in the current study are a proof of concept of nanoformulations that could be used as directed and selective drug delivery systems. The nanoformulations were liposomes functionalized with DO-24 mAb directed to a tumor-associated biomarker and contained biomimetic magnetic nanoparticles functionalized with DOXO. The size of the magnetoliposomes was <80 nm. The biomimetic magnetic nanoparticles used in this nanoformulation bound DOXO by an electrostatic interaction and became fully covered by DSPC lipids, to which DO-24 mAb was attached using a protocol optimized for these experiments. These nanoformulations were stable at physiological pH values, displaying a negative charge. Under acidic conditions, DOXO was released from the nanoformulation and exerted its cytotoxic activity when the nanoassemblies were tested on tumor cell lines. In addition, the monoclonal antibody recognizing the Met/HGFR actively drove the specific interactions of the magnetoliposomes, with cells overexpressing the cognate receptor and enhancing the delivery of the drug. The data presented here have significant potential for a directed local chemotherapy in solid tumors and can pave the way to these novel nanoformulations for future in vivo experiments.

## Figures and Tables

**Table 1:** Table reporting the compounds used for the synthesis of the nanoformulations.

Sample Name	DSCP	DSPC-PEG 2000 Malemide	PEG 2000	BMNPs	DOXO	DO-24 mAb
LP(BMNPs)	+	−	−	+	−	−
LP(DOXO-BMNPs)	+	−	−	+	+	−
mAb-LP(BMNPs)	+	+	+	+	−	+
mAb-LP(DOXO-BMNPs)	+	+	+	+	+	+

## Data Availability

The data generated during the study are available from corresponding authors on reasonable request.
